# Curated mode-of-action data and effect concentrations for chemicals relevant for the aquatic environment

**DOI:** 10.1038/s41597-023-02904-7

**Published:** 2024-01-10

**Authors:** Lena Kramer, Tobias Schulze, Nils Klüver, Rolf Altenburger, Jörg Hackermüller, Martin Krauss, Wibke Busch

**Affiliations:** 1 Helmholtz Centre for Environmental Research – UFZ, Permoserstr. 15, 04318 Leipzig, Germany; 2 RWTH Aachen University, Institute for Environmental Research, 52074 Aachen, Germany; 3 University of Leipzig, Faculty of Mathematics and Computer Science, Ritterstr. 26, 04109 Leipzig, Germany

**Keywords:** Environmental impact, Environmental monitoring, Small molecules

## Abstract

Chemicals in the aquatic environment can be harmful to organisms and ecosystems. Knowledge on effect concentrations as well as on mechanisms and modes of interaction with biological molecules and signaling pathways is necessary to perform chemical risk assessment and identify toxic compounds. To this end, we developed criteria and a pipeline for harvesting and summarizing effect concentrations from the US ECOTOX database for the three aquatic species groups algae, crustaceans, and fish and researched the modes of action of more than 3,300 environmentally relevant chemicals in literature and databases. We provide a curated dataset ready to be used for risk assessment based on monitoring data and the first comprehensive collection and categorization of modes of action of environmental chemicals. Authorities, regulators, and scientists can use this data for the grouping of chemicals, the establishment of meaningful assessment groups, and the development of *in vitro* and *in silico* approaches for chemical testing and assessment.

## Background & Summary

The intended and unintended release of micropollutants to the environment, the exposure of humans and ecosystems to those chemicals and the related potential of causing serious problems for the ecosystem and human health at various scales, has been defined as one of nine planetary boundaries^1^. More than 350,000 chemicals and mixtures thereof are in commerce^2^, and many of them are known or expected to end up in aquatic environments. Targeted screening analysis of surface waters and wastewater treatment plant effluents revealed the occurrence of up to 2000 chemicals^3–5^ in the aquatic environment. Some persistent substances, including the so-called “forever” perfluorinated compounds”, even end up far remote from production and use^6^, e.g., in high mountain^7^ or arctic^8^ areas.

Data on environmental occurrence in terms of measured environmental concentrations as well as on biological effects in terms of effect concentrations are sparse, scattered and still seldom interoperable. Nevertheless, some databases provide such information; for example, the NORMAN network collects chemical concentrations measured in European monitoring campaigns^9^, and the ECOTOXicology Knowledgebase (ECOTOX) developed by the US Environmental Protection Agency (US EPA) provides toxicity data for environmental species^10^. Such data is required for hazard and environmental risk assessment.

In the ideal case, experimental toxicity data is available for all chemicals and species present in the (aquatic) environment. In reality, scarcity of the relevant data for many chemicals often limits the risk prediction power. To fill gaps in the (eco)toxicity data, quantitative structure-activity relationship (QSAR) models are utilized. Lacking experimental data, these models estimate a chemical’s bioactivity or toxicity based on its structure^11^. While classical QSAR models are typically based on linear regression models to predict chronic or acute toxicity of chemicals from the calculated physico-chemical properties^12,13^, many of the modern QSARs apply machine-learning approaches to estimate effect concentrations based on molecular descriptors^14–16^. Furthermore, structure-based classification schemes exist to categorize chemicals based on their mode of toxic action (MoA). However, available tools to predict such MoA, for instance, the Verhaar scheme^17^, the EPA ASTER QSAR application^18^, and the EPA MOAtox database^19^, are limited in their ability to provide consistent predicted MoAs for a wide range of chemicals^20^.

Due to the current limitations in data availability and in the predictability of potential long-term effects with short-term acute toxicity testing, as well as the time lag and costs associated with chronic toxicity testing using animals, risk assessment is about to change towards evidence-based and integrated assessments considering mechanistic knowledge and evidence on a chemical’s mechanism of toxic action derived from *in vitro* bioassays or read across approaches^21–23^. The adverse outcome pathway (AOP) concept, introduced by Ankley *et al*.^24^ and implemented by the OECD^25^ helps to organize such knowledge from any kind of chemical-biological interaction studies and to inform such evidence-based approaches. Established AOPs provide mechanistic information, e.g., from a chemical binding to a biological receptor molecule exerting physiological events to the development of an adverse outcome. With this, AOPs contain information on a chemical’s MoA and offer cross-species considerations as well as read across options^26^. Using mechanistic information for grouping of chemicals according to their, e.g., similar biological MoA, has been proposed for the performance of risk assessment by EFSA^27^ and ECHA^28^. Thereby, EFSA defines a biologically plausible sequence of events in an organism leading to an observed effect as MoA. It refers to the major steps leading to an adverse health effect following interaction of the chemical with biological targets, but does not imply full understanding of the mechanism of action at the molecular level^29^.

In 2016, Busch *et al*.^3^ researched mechanisms of action of and knowledge on MoAs for more than 400 environmentally relevant chemicals and showed that diverse pesticides, pharmaceuticals, and industrial chemicals can have the same or similar MoAs, for example, acting on the nervous or endocrine system of vertebrates. This is of relevance, especially for non-target organisms that are unintended co-exposed to mixtures of such chemicals in the aquatic environment. Chemical regulation and authorization, however, does, in most cases, not consider co-exposures and is separated into regulatory silos according to the intended chemical use domain. Recent efforts discuss the implementation of a mixture assessment factor^30^ or the development of cumulative assessment groups of chemicals^27^ to cope with this issue. MoAs or mechanistic information are, however, not considered yet within these recent environmental mixture risk assessment considerations.

Here, we aimed to extend the previous list of 426 environmentally relevant chemicals to a comprehensive list of 3,387 compounds and provide information on their MoAs as well as on their toxicity to support environmental risk assessment. Therefore, we undertook the effort to research, merge, curate, and provide data on biological MoAs, as well as curated effect concentrations for each compound for three biological quality elements (BQE) of the European Water Framework Directive, namely algae, crustaceans, and fish (https://www.eea.europa.eu/themes/water/european-waters/water-quality-and-water-assessment/water-assessments/quality-elements-of-water-bodies). The data should be provided in a FAIR format to enable standardization, comparability, reproducibility, and acceleration for ecotoxicological hazard and risk assessment^31^.

Based on information from databases, lists of regulatory directives, monitoring projects, and respective chemical suspect lists, we compiled a curated list of 3,387 compounds of freshwater environmental occurrences e.g.^32–36^. We researched all chemicals on this list for their use and product group, their biological MoAs, and compiled ecotoxicity data ready to be used in environmental risk assessment based on data from the US EPA ECOTOXicology Knowledgebase^10^ (ECOTOXDB) and QSAR predictions. Out of all 3,387 compounds, 2,890 were identified as parent substances, 374 as transformation products (TP), and 96 as both, parent and TP. Only in a few cases (27 chemicals) of mainly industrial chemicals (e.g., 3-dodecylbenzene-1-sulfonic acid.), such a classification could only be assumed, and was, therefore, not assigned (Tables A, B, and C, available on Zenodo)^37^. Further details on the curated use group classifications, the MoA categories, and the toxicity data for all compounds are explained and summarized below.

## Methods

### Use groups, mode of action and accompanying information

#### Overall strategy

For each compound in the dataset, a systematic search in different data sources and databases was performed. All information was obtained from the following databases listed in Table 1.

**Table 1:** List of databases for the research on the use of chemicals and information about modes of action.

Database Name	Description	URL	Access dates	Ref.
Pesticide Properties DataBase (PPDB)	A comprehensive relational database of pesticide chemical identity, physicochemical, human health and ecotoxicological data	http://sitem.herts.ac.uk/aeru/ppdb/en/search.htm	Jan 2021 – Apr 2023	^38^
Bio-Pesticides DataBase (BPDB)	A comprehensive relational database of data relating to pesticides derived from natural substances	http://sitem.herts.ac.uk/aeru/bpdb/search.htm	Jan 2021 – Apr 2023	^59^
Veterinary Substances DataBase (VSDB)	A comprehensive relational database of physicochemical and toxicological data for veterinary substances	http://sitem.herts.ac.uk/aeru/vsdb/search.htm	Jan 2021 – Apr 2023	^59^
Insecticide Resistance Action Committee (IRAC): Mode of Action classification	A global scheme on the mode of actions and target sites of acaricides and insecticides	https://irac-online.org/mode-of-action/classification-online/	Jan 2021 – Apr 2023	^59^
Herbicide Resistance Action Committee (HRAC): Global herbicide classification lookup	A global scheme on the mode of actions and target sites of herbicides	https://hracglobal.com/tools/classification-lookup	Jan 2021 – Apr 2023	
Fungicide Resistance Action Committee (FRAC): Mode of Action classification	A global scheme mode of actions and target sites of fungicides	https://www.frac.info/fungicide-resistance-management/by-fungicide-common-name	Jan 2021 – Apr 2023	
Drugbank	A comprehensive, online database containing information on drugs and drug targets	https://go.drugbank.com/	Jan 2021 – Apr 2023	^60^
PubChem	A comprehensive collection of freely accessible chemical information	https://pubchem.ncbi.nlm.nih.gov/	Jan 2021 – Apr 2023	^41^
Wikipedia	A free online encyclopedia	https://en.wikipedia.org/wiki/Main_Page	Jan 2021 – Apr 2023	
Human metabolome database (HMDB)	An electronic database containing detailed information about small molecule metabolites found in the human body	https://hmdb.ca/	Jan 2021 – Apr 2023	^61^
Chemical and Products Database (CPDat)	A database containing information on usage or function of consumer products and chemicals	https://comptox.epa.gov/dashboard/ https://epa.figshare.com/articles/dataset/Chemical_and_Product_Categories_CPCat_database/7871537	Jan 2021 – Apr 2023	^62^

We did not use specific search terms within those databases but collected all information on MoA and use that was available for the respective compound. For compounds with uncertain or limited information available, results of a literature research were included to fill as many remaining gaps as possible. There, we searched the web of Science and PubMed databases and used the compound name in combination with “toxicity” or “mode of action”. Results of these searches were not systematically stored, but relevant information transferred to our collection. Data were retrieved between January 2021 and April 2023. All collected data were sorted and categorized at higher levels: based on the results, each compound was assigned as a parent or transformation product; assigned to a specific use group; and if possible, to a mode of action and target or non-target taxa, as pointed out in detail below. Overall, we followed a step-wise approach where all information for a respective compound was collected first, and sorted and annotated according to our systematic and standardized broad categories of use groups and MoAs in a second step. Finally, we curated all further information and developed standardized terms for detailed use information as well as for specific MoA information, although this was challenging and some chemicals remained with individual descriptions (Tables A and C, available on Zenodo)^37^.

#### Use group classification

Based on the collected information, all compounds were assigned either as parent compound or as transformation product (TP) in column “parent_or_TP” (Tables A and C, available on Zenodo)^37^. Respective parent compound(s) are listed for all TPs in column “TP_of”. In the context of this dataset, TPs comprise both, metabolites and environmental degradation products. Some chemicals are both, they occur as TP of another compound, but are also parent compounds in another context. These chemicals were labeled as ‘parent + TP’. In our statistical analyses, we counted them as parent substances. In the next step, the compounds were grouped according to their use into eight categories: (1) Industrial Chemical, (2) Pesticide, (3) Biocide, (4) Pharmaceutical, (5) Drug of abuse, (6) Natural, (7) Food additive, and (8) Metal (column”use_group”). The groups “drug of abuse” and “biocide” are listed as separate use groups in the dataset but were included into the use group categories of “Pharmaceutical/Drug of abuse” and “Pesticide/Biocide”, respectively, in the analyses and figures. As “drugs of abuse” we defined psychoactive drugs that are not used as pharmaceuticals such as, e.g., Nicotine, or 3,4-Methylenedioxyamphetamine (MDA). Multiple use groups can be assigned to a compound depending on its range of applications.

**Fig. 1:**
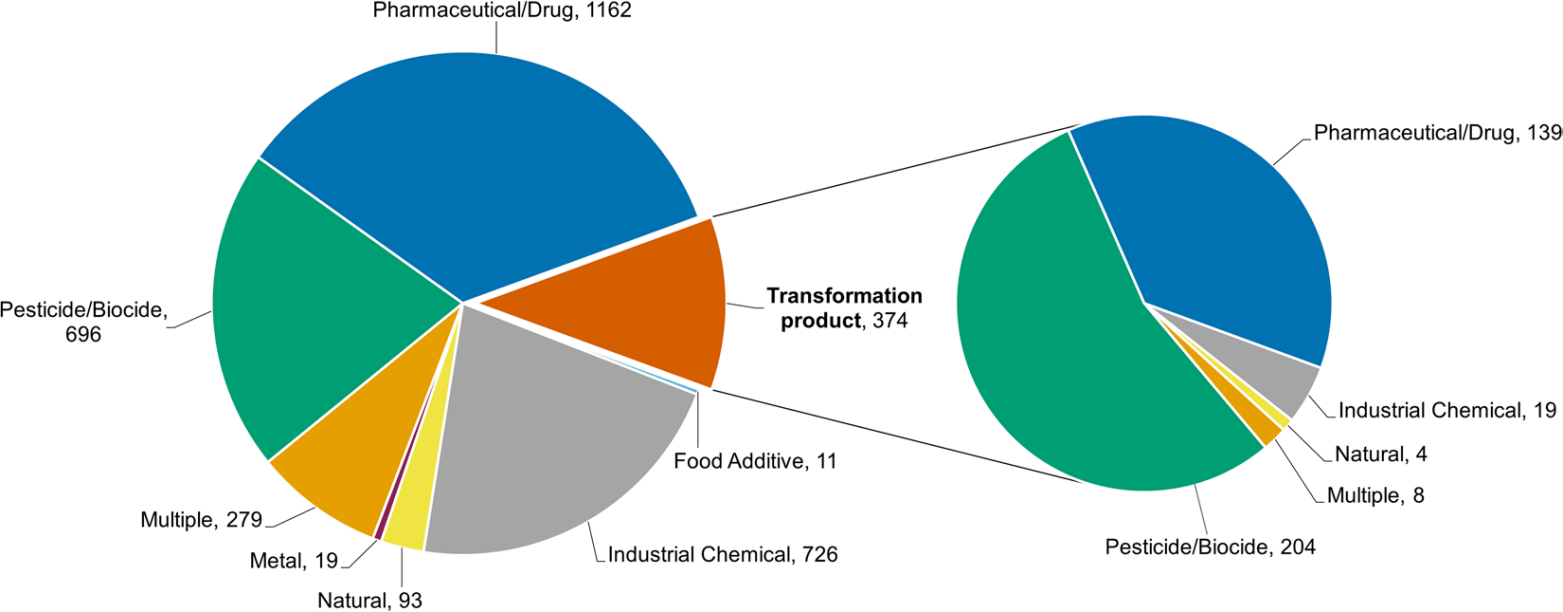
Number of compounds in the dataset categorized as ‘parent’ or ‘parent + TP’ (left) and transformation products (right) per use group category.

The category of pharmaceuticals and drugs is the most common in the dataset with 1,162 compounds and additional 139 TPs, followed by pesticides and biocides with 696 compounds and 204 TPs, industrial chemicals with 726 and 19 TPs, naturally occurring compounds with 93 and 4 TPs, metals with 19 compounds and food additives with 11 compounds (Fig. 1). 279 compounds (8 TPs) were assigned to more than one use group and are summarized as multiple use compounds in the graphs. These include food additives which predominantly have an additional application, e.g., in the industrial sector as fragrance. Moreover, many naturally occurring compounds such as alkaloids or hormones are frequently applied as pesticides, pharmaceuticals or in industrial applications.

Detailed information on the application and usage of all compounds in each use group category were collected and added to the table in additional columns (“use_group_details” and “additional_use_info”). Pesticides were further categorized systematically into insecticides (I), acaricides (A), herbicides (H), fungicides (F), and other specific use groups, e.g., molluscicides and nematicides. Detailed application areas of pharmaceuticals were included and annotated according to their area of action, ranging from antibiotics over contraceptives to psychotropic or radioactive agents, just to mention a few examples. This listing contains more than 100 terms and was curated based on the retrieved information, but does not necessarily consider medical term standards. Moreover, specific applications of industrial chemicals are listed and respective terms were taken from the collected information or curated by us. If chemicals belong to different use groups, this is indicated in the “use_group” column where all broad application domains are listed, separated with “,”. In the “use_group_details” applications in different sectors, e.g., as industrial chemical and insecticide, are listed with “ + ” while multiple uses within a sector are separated with “/”. This listing is far from being comprehensive but covers the most common use domains for the investigated chemicals, whereas “most common” was a subjective decision by the curator based on the retrieved information. All abbreviations used in the mentioned columns of Table C are explained next to the column header explanations in Table A (available on Zenodo)^37^. The “additional_use_info” column contains additional non-systematic and non-standardized information on the use of the chemical.

Most industrial chemicals are used for multiple applications, e.g., as colorants, fragrances, plasticizers, additives, flame retardants, solvents, reagents, or intermediates in the manufacture of other products. Furthermore, 105 compounds within the industrial chemicals group have an application as personal care (PC) products such as stabilizer, surfactant or UV filter in sunscreen products e.g., benzophenone-3. Compounds that are found in diesel exhaust, cigarette smoke or which are generally released in combustion processes of natural compounds are summarized as combustion products (59 compounds) within the group of industrial chemicals. Contrary to the multiple applications of industrial chemicals, pesticides are more specific in their use. Within the group of pesticides, 126 (40 TPs) compounds are assigned as pure insecticides, and 99 compounds (10 TPs) as both, insecticide and acaricide. The dataset includes 251 (83 TPs) herbicides, 124 (41 TPs) fungicides, and 73 compounds (26 TPs) with multiple applications or a specific use, e.g., as nematicide, molluscicide, avicide, or as herbicide safener. 80 compounds are considered obsolete, and 49 pesticides are also used as veterinary substances. Pure biocides are rare because most compounds with biocidal action also serve as industrial chemicals, or have an application in the agricultural sector as pesticide. The group of pharmaceuticals can be further distinguished into various detailed use groups, with psychotropic drugs, antibiotics, antihypertensive, antihistamine, and anti-inflammatory drugs being the most prominent. There is a large overlap between the use groups of pharmaceuticals and drugs of abuse, e.g., for doping agents used in sports. Otherwise, the use group of drugs of abuse contains mostly cannabinoids and stimulating, psychedelic or hallucinogenic substances. Finally, even with additional literature research efforts, 27 compounds could not be assigned to a specific application sector and remain with an unknown use group. Consequently, these are the ones for which an assignment to parent and TP was not possible.

#### Mode of action

The amount of information available on MoAs varied significantly between use groups and chemicals. MoA information covers molecular targets (e.g., action on a specific receptor), a pathway (e.g., photosynthesis inhibition), and/or general information about a disturbance (e.g., endocrine disruption).

Based on the collected data and information and on our previous work^3,4^, we aimed to provide systematic and harmonized broad MoA categories but also additional and more specific information about the biological actions of the chemicals. Therefore, we started to note down whether there are target and/or known non-target species for the respective chemicals, their biological molecular targets and/or specific ways of action as well as additional MoA information. We sorted the retrieved information, which was available for more than half of the compounds, and established harmonized terms for specific MoAs and the broad MoA categories systematically. We grouped chemicals that act on the same target or the same biological signaling pathway into a common “MoA_specific” also considering terms established by the HRAC, IRAC and FRAC databases. Different specific MoAs that could be assigned to a broader biological system, such as, e.g., the nervous system or the endocrine system, were then grouped into common “MoA_broad” categories (Table C, columns “MoA_broad” and “MoA_specific”, available on Zenodo)^37^. Abbreviations for biological molecules used within these terms, such as, e.g., AChE for acetylcholine esterase, are explained in the following columns that contain information on molecular targets and further MoA details. The contents of these additional columns were only partly harmonized (Table C, columns “molecular_target” and “further_MoA_info”, available on Zenodo)^37^. Finally, many compounds act on multiple molecular targets and can be assigned to more than one specific or broad MoA group. In these cases, all MoAs are listed with “,” in the respective columns.

**Fig. 2:**
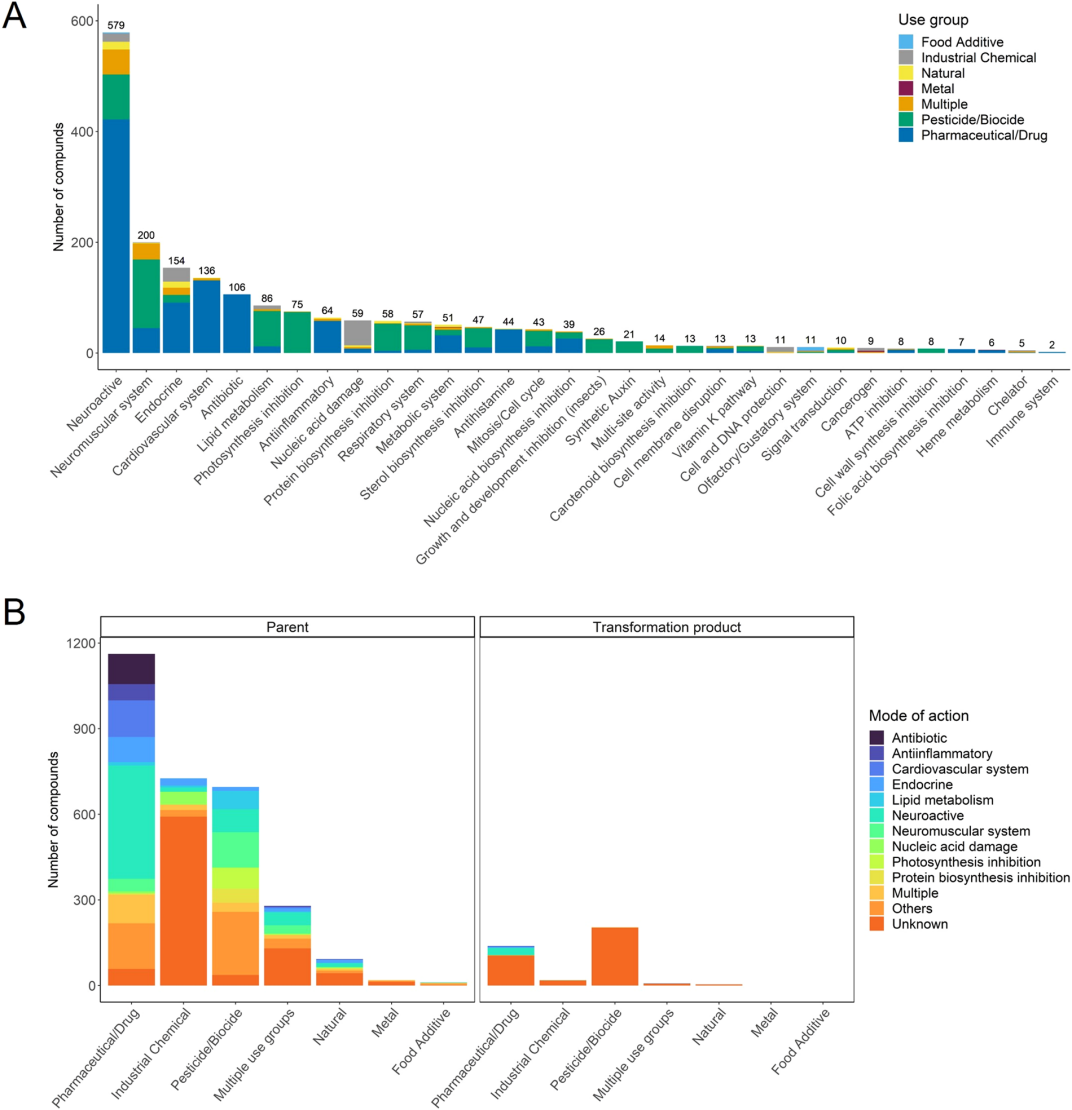
(**A**) Curated broad mode of action (MoA) categories with the number of assigned compounds in the dataset; colors indicate the different use group categories, chemicals with multiple or unknown MoA broad are not considered in this figure; (**B**) Number of chemicals distributed across the ten most common broad MoA categories per use group in our dataset, among the parent compounds (left), and the transformation products (right).

MoA information could be retrieved for 2,172 compounds (64%) for at least one of the three levels (broad, specific, or molecular target) (Table S3). Of those, 1,975 were assigned to one of the 32 broad MoA categories, 174 to two or more categories and 1,238 (including 337 TPs) could not be assigned to a broad MoA category. Considering compounds with one broad MoA, chemicals with a neuroactive mode of action build the most prominent group (579 compounds), followed by compounds acting on neuromuscular (200), endocrine (154), and cardiovascular systems (136) as exemplarily shown in Fig. 2A. While, for example, the MoA category ‘Antibiotics’ and ‘Synthetic auxins’ contain chemicals of only one-use group, pharmaceuticals and pesticides, respectively, most MoA categories contain chemicals of different use groups. 15 broad MoA categories could be discriminated within the group of industrial chemicals, led by the categories ‘Nucleic acid damage’ and ‘Endocrine’ with 55 and 38 chemicals, respectively. 25 broad MoA categories were identified among the pharmaceuticals/drugs, with ‘Neuroactive’ (458) and ‘Cardiovascular system’ (150) being those with the most assigned chemicals. 23 MoA broad categories were identified for the pesticides/biocides, with the largest number of chemicals known to act on the ‘Neuromuscular system’ (124) and as ‘Neuroactive’ compounds (85). Only for 10% of all TPs, a broad MoA category could be assigned (Fig. 2B). Although a transformation product is sometimes the actual acting chemical, MoA information is in most cases described for the parent compound. Therefore, details on the biological action of TPs are widely lacking. We did assign the MoA of a parent compound also to its TP only in cases, where the respective action of the TP was clearly indicated in a respective information sources, which is also given Table C (column “source”, available on Zenodo)^37^.

**Fig. 3:**
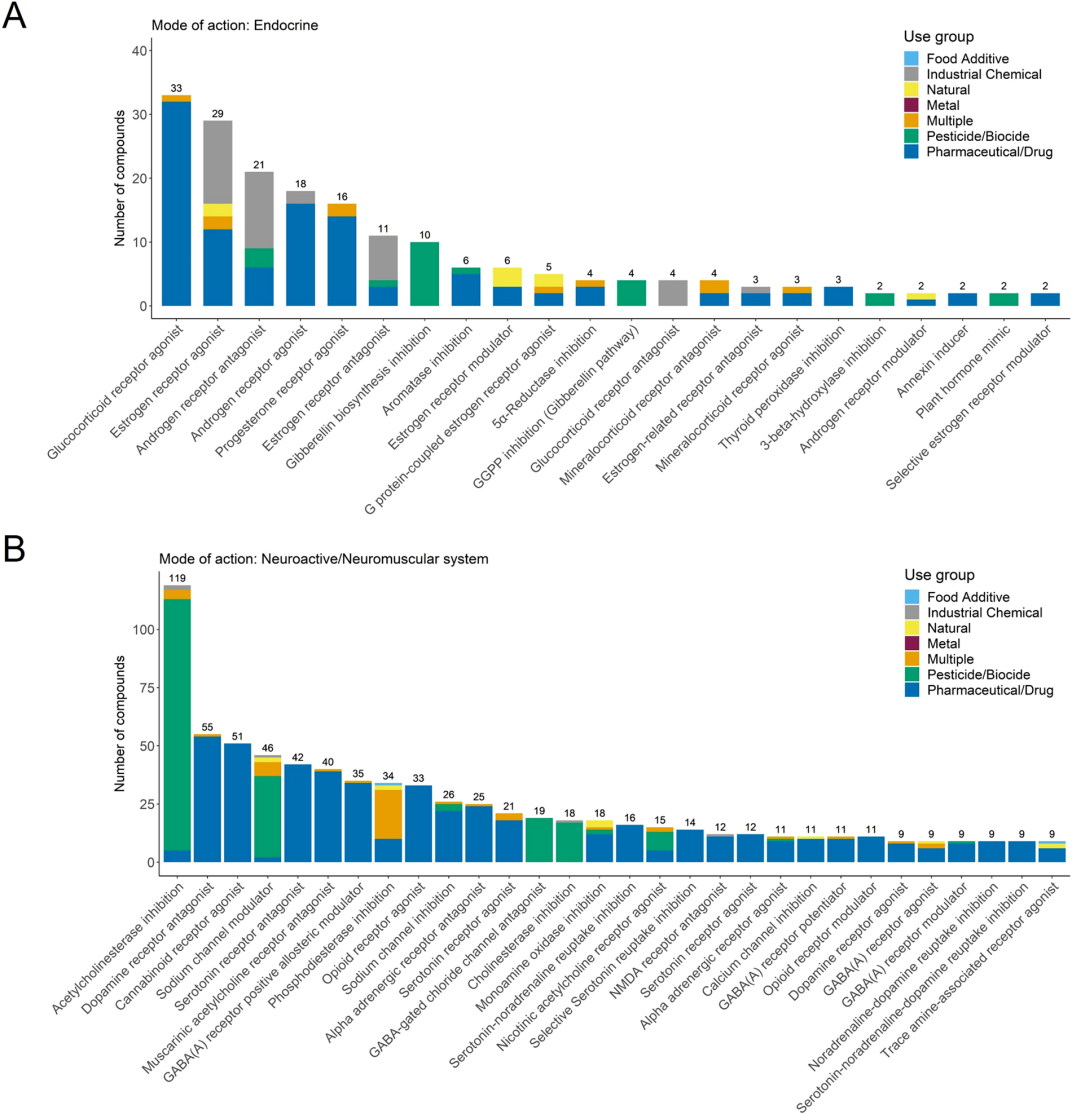
Number of chemicals assigned to specific MoAs within two broad MoA categories in the dataset (**A**) ‘Endocrine’ and (**B**) ‘Neuroactive’ and ‘Neuromuscular system’ colored according to use groups. MoA specific categories with less than two chemicals in (**A**) and nine chemicals in (**B**) are, respectively, not shown.

Although most chemicals could be assigned to one broad MoA category, more specific considerations showed that they still can act on multiple molecular targets in the same or across species. Up to eight molecular targets or specific MoAs were found per compound. There are considerable differences between the numbers of specific MoAs or molecular targets within one broad MoA category. ‘Endocrine’ acting chemicals were found across all use groups (including industrial chemicals). This broad MoA category contains 48 specific MoAs, with action on the glucocorticoid, androgen, and estrogen receptors being the most abundant ones (Fig. 3A). The broad MoA category ‘Endocrine’ accounts for all organisms which includes action on the hormone system of animals or humans, e.g., androgen or estrogen receptors, as well as the plant hormone syntheses, e.g., the inhibition of the gibberellin biosynthesis. This indicates that the broad MoA categories help to summarize the biological actions exerted by chemicals in the environment, but for detailed analysis or species-specific assessments the specific MoAs need to be considered. Indeed, single MoA broad categories are overlapping, e.g., the category ‘Neuromuscular system’ is related to the category of ‘Neuroactive’ compounds. In Fig. 3B it is shown that these two categories alone comprise more than 100 specific MoAs. Again, it becomes obvious that some seem to be use group-specific (e.g., serotonin receptor antagonists are all pharmaceuticals) while other specific MoAs contain chemicals of different use groups (e.g., the specific MoA with the largest number of chemicals in this category: ‘Acetylcholinesterase inhibition’).

Finally, multiple pharmaceuticals, e.g., cortisol, serve as pro-drugs and are only active at a specific target when transformed to their active metabolites. Even though the prodrug itself is inactive, we decided to include the MoA of the respective active metabolite in the dataset. Furthermore, a unique assignment of a molecular target to a single broad MoA category is often challenging. For instance, cannabinoids act on the endocannabinoid system and are declared as neuroactive in the dataset, but effects on the immune system have also been proposed for the cannabinoid receptor 2. Therefore, the classification of broad MoA categories serves as a guidance and can be further discussed and developed in the future.

### Target/non-target taxa and additional information

Data about action on target species (e.g., insects in the case of insecticides) and non-target species (e.g., other aquatic species or humans) were included into the dataset in case such information occurred during the curation. However, we did not systematically search for non-target effects and information of all compounds. A target taxon could be assigned for pesticides, e.g., insects, plants, fungi, or nematodes as these chemicals are designed, respectively. Moreover, for most pharmaceuticals and drugs of abuse, humans can be declared as target species, while in the case of veterinary substances, the target group can be extended to mammals or even vertebrates. The target taxa of antibiotics, antiviral and antifungal pharmaceuticals were specified as bacteria, viruses and fungi in humans, respectively. No target species was determined for natural compounds, food additives, metals or industrial chemicals, with a few exceptions, such as disinfectants or antifungals with target taxa of bacteria or fungi.

While MoAs of pesticides and pharmaceuticals are intentionally designed for specific target taxa, their toxicological MoA on vertebrates as non-target species might be entirely different. For most of the chemicals, little information about the mechanisms of action in non-target species was identified. However, in cases where such information was found during our research, alerts for non-target species were included into the dataset. Risks of pesticides for non-target species were retrieved from the PPDB^38^ database, risks of potential carcinogenic compounds for humans were retrieved from the International Agency for Research on Cancer (IARC)^39^ of the World Health Organization, and information on endocrine disruptors were retrieved from the endocrine disruptor lists of ECHA^35^. Therefore, Table C also contains the columns “nontarget_taxa” and “nontarget_taxa_alert” available on Zenodo^37^.

### Chemical data

#### Data compilation and retrieval

The list of chemicals was compiled based on existing collections of known environmental contaminants and amended by chemical information retrieved from US EPA Chemical Dashboard^40^ and PubChem^41^. The data were linked by unequivocal identifiers (i.e. InChiKey, PubChem CID, and DTXSID).

#### Curation of chemical structures

To improve the quality of the chemical structures (i.e., SMILES) and QSAR modeling, curation of all structures was applied. It has been proven that deploying the pure and neutralized chemical structures enhances the quality of machine learning based QSAR predictions^15,42^. The curation included, for example, the canonicalization of the SMILES code, the desalting, de-aromatization, removal of stereochemistry, solving hyper valency, and other transformations to generate QSAR-ready SMILES described elsewhere utilizing OPERA 2.7^15^.

#### Prediction of log S_w_ values

The water solubility at 25 °C of all included was predicted applying OPERA 2.7 based on QSAR-ready SMILES^42^. OPERA exports the values in csv format. The unit of log S_w_ is mol/L. The S_w_ in mg/L was calculated using the average molecular mass in g/mol. The solubility was needed to estimate the solubility domain class of the ecotoxicity data described below.

### Ecotoxicity data

#### US EPA ECOTOXicology Knowledgebase data

Environmental risk assessment requires exposure as well as effect concentrations and is often challenged by the variety of experimentally determined effect concentrations that differ between species, endpoints, and experimental settings. In this study, we aimed to provide one value per compound and BQE (algae, crustaceans, and fish according to the WFD), derived from all available data in the ECOTOXDB^10^ using carefully selected and developed criteria that ensure that comparable data is selected and merged, and a robust and representative effect concentration for each respective species group is derived. The whole procedure of data retrieval and processing was performed using the R package REcoTox^43^ version 0.4.1 (https://github.com/tsufz/REcoTox/tree/0.4.1). The software and workflow is described briefly below and in detail in the vignette document^44^.

#### Data retrieval, processing, and curation

The ASCII file version *ecotox_ascii_03_10_2022.zip* of the ECOTOXDB^10^ was downloaded from US EPA website (https://cfpub.epa.gov/ecotox). The database files were unzipped and the tables tests, results, species, chemicals, and references were selected for further processing. Data from the selected tables were joined and further filtered by the IDs test_id, cas_number, species_number, and reference_number, and under consideration of the following criteria:

- type of dosing (only water-based dosing was considered, e.g., mg/L); dosing_group = “water_concentration“
- time unit of endpoint (e.g., d for days, h for hours); duration_d = c(“d“, “dph“, “dpf“); duration_h = c(“h“, “ht“, “hph“, “hpf“, “hv“)
- duration range of the exposure (e.g., minimum and maximum hours); duration_m = mi; min_d = 0; min_h = 0; min_m = 0; max_d = 5; max_h = 120; max_m = 7200
- species (e.g., species group (ecotoxgroup) such as *algae*, habitat, subsets of species like standard species); ecotoxgroup = c(“Algae“, “Crustacean“, “Fish“); habitat = “Water“; species_selection = “all“ for “Algae“; species_selection = “standard_test_species“ for “Crustacean“ and “Fish“
- effects and measurements; effects = c(“MOR“, “GRO“, “POP“, “REP“, “MPH“, “DEV“) for “Algae“ and “Fish“; effects = c(“MOR“, “GRO“, “POP“, “REP“, “MPH“, “DEV“, “ITX“) for “Crustacean“

The definitions of the ECOTOXDB terms are detailed elsewhere (ECOTOX Help. https://cfpub.epa.gov/ecotox/help.cfm?sub=term-appendix).

According to Busch *et al*.^3^, the selected endpoints were cover the whole distribution of the concentration-effect-relationships. This includes all effect or lethal levels recorded in the ECOTOXDB namely: effect concentrations - EC_1–99_, effective doses - ED_1–99_, effect levels - EL_1–99_, inductive concentrations - IC_1–99_, lethal concentrations - LC_1–99_, lethal doses - LD_1–99_, lethal levels - LL_1–99_, lethal thresholds - LT_1–99_, lowest observed effect concentrations - LOEC, and lowest observed lethal concentrations - LETC.

Afterwards, the different dosing units were standardized to mg/L. In cases where mol/L are used in the database, the table ecotoxgroup_mol_weight.csv was applied to recalculate concentrations to mg/L.

In this study, the percentile value was set to 0.05 (quantile = 0.05) to calculate the 5^th^ percentile of the included effect data. Additionally, the average, the geometric average, the median, the minimum, and the maximum effect concentrations were calculated and rounded to four significant digits. The estimated values can exceed the solubility of the single compounds. In older studies, often the nominal concentration was reported without measurement of the real concentrations in the bioassays. Some reported values might be thus overestimated. To mitigate the risk of false positive results, the so-called solubility domain was calculated according to the ideas realized in ChemProp^45^ (https://www.ufz.de/index.php?en=34593).

The solubility domain has three classes:

$$E{C}_{x}\le {S}_{w}$$

$${S}_{w} < E{C}_{x}\le 1{0}^{5}\times log\left({S}_{w}\right)$$

$$1{0}^{5}\times log\left(S\right) < E{C}_{x}\le 1{0}^{10}\times log\left({S}_{w}\right)$$

$$E{C}_{x} > 1{0}^{10}\times {S}_{w}$$

Where *EC*_*x*_ is the respective ecotoxicity value and *S* is the solubility value in mg/L. The basis of *log* (*S*) is 10. The classes of the solubility domain were calculated for each of the aggregated values (e.g., geometrical mean) and amended to the results table. Furthermore, all raw ecotoxicity values, endpoints, effects, measurements, species, durations, and reference IDs are collapsed in one single field per category for later review.

#### Prediction of effect concentrations

Measured effect concentrations were successfully derived and curated for a maximum of 25% of all compounds. Therefore, we additionally predicted effect concentrations for all chemicals for which the respective QSAR models^15,16,42^ were applicable. It was not possible to predict values for inorganic and metal-organic compounds, and complexes because existing QSAR models do not handle such structures. In addition, mixtures were excluded. The VEGA QSAR models^16^ (https://www.vegahub.eu/portfolio-item/vega-qsar) were applied to QSAR-ready SMILES, including these QSAR models:

- Algae acute [EC50] toxicity model (IRFMN) v. 1.0.1
- *Daphnia magna* acute [EC50] toxicity model (IRFMN) v. 1.0.1
- Fish acute [EC50] toxicity model (IRFM) v. 1.0.1

The IRFMN models report the toxicity values in -log(mmol/L). In addition, an a-dimensional predicted value is reported. Since the employed version of the models contained a software bug related to molecular weight estimation, the a-dimensional value was used to calculate correct predicted values (email correspondence with IRFMN). The correction requires a box-cox transformation of the a-dimensional values of the algae and fish models utilizing equations

$$E{C}_{50,algae}\left[mmol/L\right]={\left(E{C}_{a-dimentional,algae}\times 0.07+1\right)}^{\frac{1.0}{0.07}}$$

$$E{C}_{50,fish}\left[mmol/L\right]={\left(E{C}_{a-dimentional,fish}\times 0.11+1\right)}^{\frac{1.0}{0.07}}$$

### Summary of ecotoxicity dataset

**Fig. 4:**
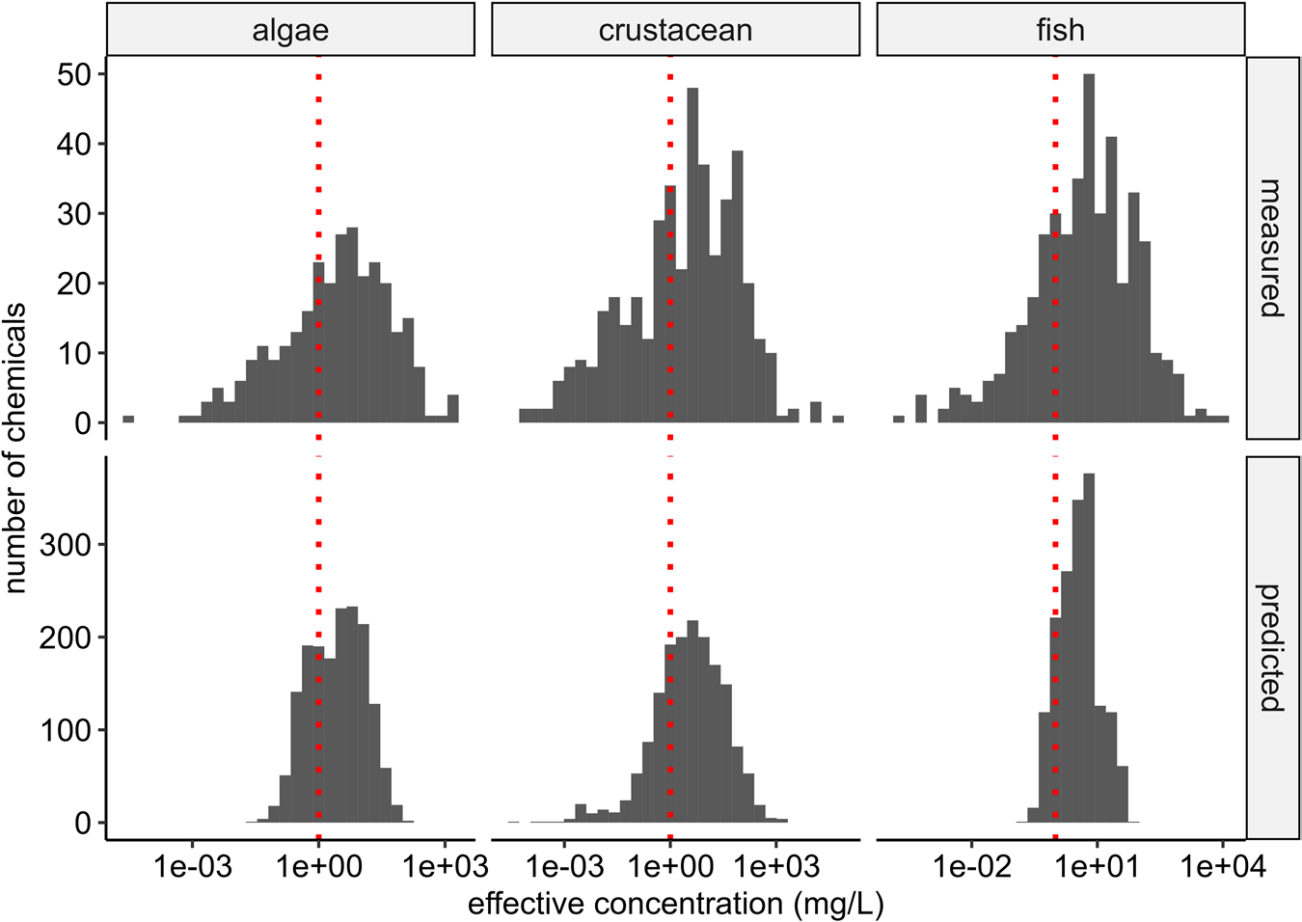
Distributions of measured and predicted effect concentrations for the three BQE (algae, crustacean, and fish) according to the data availability listed in Table 1.

The distributions of measured (retrieved from the ECOTOXDB) and QSAR-predicted effect concentrations for the biological quality elements (BQE)^46,47^ algae, crustaceans, and fish are shown in Fig. 4 and Table 2. The measured values, which were considered in this dataset, cover 586 (17%), 858 (25%), and 855 (25%) out of the 3,387 chemicals included here, respectively, for algae, crustacean, and fish. The experimental data is based on 6,156, 9,760, and 19,416 data points with a mean of 5 ± 3, 4 ± 3, and 7 ± 4 data points per chemical, respectively, for algae, crustacean, and fish (Table 3).

**Table 2:** Numbers and percentage of chemicals for which measured and predicted effect concentrations could be derived (according to the selection criteria of this study (# = numbers)).

BQE	Algae	Crustacean	Fish
**Measured (#)**	586	858	855
**Measured (%)**	17.3	25.3	25.3
**Predicted (#)**	3,318	3,318	3,318
**Predicted (%)**	98	98	98

**Table 3:** Total number, geometrical mean, and geometrical standard deviation of data points per chemical available in the ECOTOXDB and considered in this study.

BQE	Algae	Crustacean	Fish
**Total number of data points**	6,156	9,760	19,416
**geoMean (data points per chemical)**	5	4	7
**geoSD (data points per chemical)**	3.1	3.5	4.2

While the applied QSARs predict to which extent the processed chemical structure covers the application domains of the models, the quality and accuracy of the experimental data is difficult to discover. The QSAR derived application domain classes assigned to each chemical included in this dataset are listed in the respective Tables D-F (available on Zenodo)^37^.

### Data compilation

The final tables were processed using an R script. The different tables are linked by an internal identifier (ID). The dataset includes mixtures or compounds without InChiKeys, PubChem CIDs and/or DTXSIDs, and thus no other unequivocal identifier was applicable.

## Data Records

All data is available on ZENODO^37^ (10.5281/zenodo.10071824). The ZENODO repository contains the following six tables in csv format and one table in xlsx format, containing all six csv files. The first table contains explanations for all columns of the dataset:

Table A_column_descriptions_for_Tables B to F.csv

This table contains all column headers of the following tables B to F and respective explanations and descriptions.

Table B_chemical_information.csv

Table B contains chemical names and common identifiers, such as CAS numbers, PubChem IDs, DTXSIDs, InCHI and Smiles codes for all 3387 chemicals considered in this study.

Table_C_use_groups_and_mode_of_action_information_for_chemicals.csv

Table C contains the collected and curated information on the usage domains of all chemicals and their biological modes of action. The curation process and all data sources are described in the methods part.

Table_D_ecotoxicity_data_algae.csv

Table D contains measured and predicted acute toxicity data for algae species curated and derived for all chemicals as described in detail in the methods part.

Table_E_ecotoxicity_data_algae.csv

Table E contains measured and predicted acute toxicity data for the species group of crustaceans curated and derived for all chemicals as described in detail in the methods part.

Table_F_ecotoxicity_data_fish.csv

Table F contains measured and predicted acute toxicity data for fish species curated and derived for all chemicals as described in detail in the methods part.

Table_X_Tables_A_to_F_chemicals_MoAs_ecotoxdata.xlsx

Table X is provided as additional service and contains all csv tables in an Excel spreadsheet.

## Technical Validation

### Toxicity data and chemical solubility

Using and curating experimental data is always biased by the availability of the data itself. There are different reasons for variations in the availability of experimental data for different chemicals: i) not all chemicals have been tested in bioassays, ii) some chemicals were only tested in a single study, in a single bioassay, with only one species, or with one specific exposure setting, iii) newer chemicals have been screened less often than older (legacy) compounds. For example, this dataset included 475 individual data points for the (legacy in Europe) herbicide atrazine tested in an algae bioassay, but only one datapoint for the herbicide mecoprop, which is currently used.

For filling such data gaps, we used QSARs that are state-of-the art and compliant with the recommendations of the Organization for Economic Co-operation and Development (OECD)^48^. The QSAR predicted data cover 3,318 (98%) compounds for the three species groups. In theory, the property of each chemical could be predicted using QSARs. However, QSARs do not cover all structures or compounds (e.g., organometallic or inorganic compounds cannot be predicted) and some included substances in our research are mixtures which are as well not covered by our QSAR analysis. The distribution of effect concentrations in Fig. 4 are broader for the experimental data compared with the predicted values by one to two log orders of magnitude, while the median effect concentrations across all chemicals are similar between the measured and predicted data.

The quality of the experimental and computational ecotoxicity data was validated using a solubility domain classification to identify and annotate possible outliers in the dataset.

**Fig. 5:**
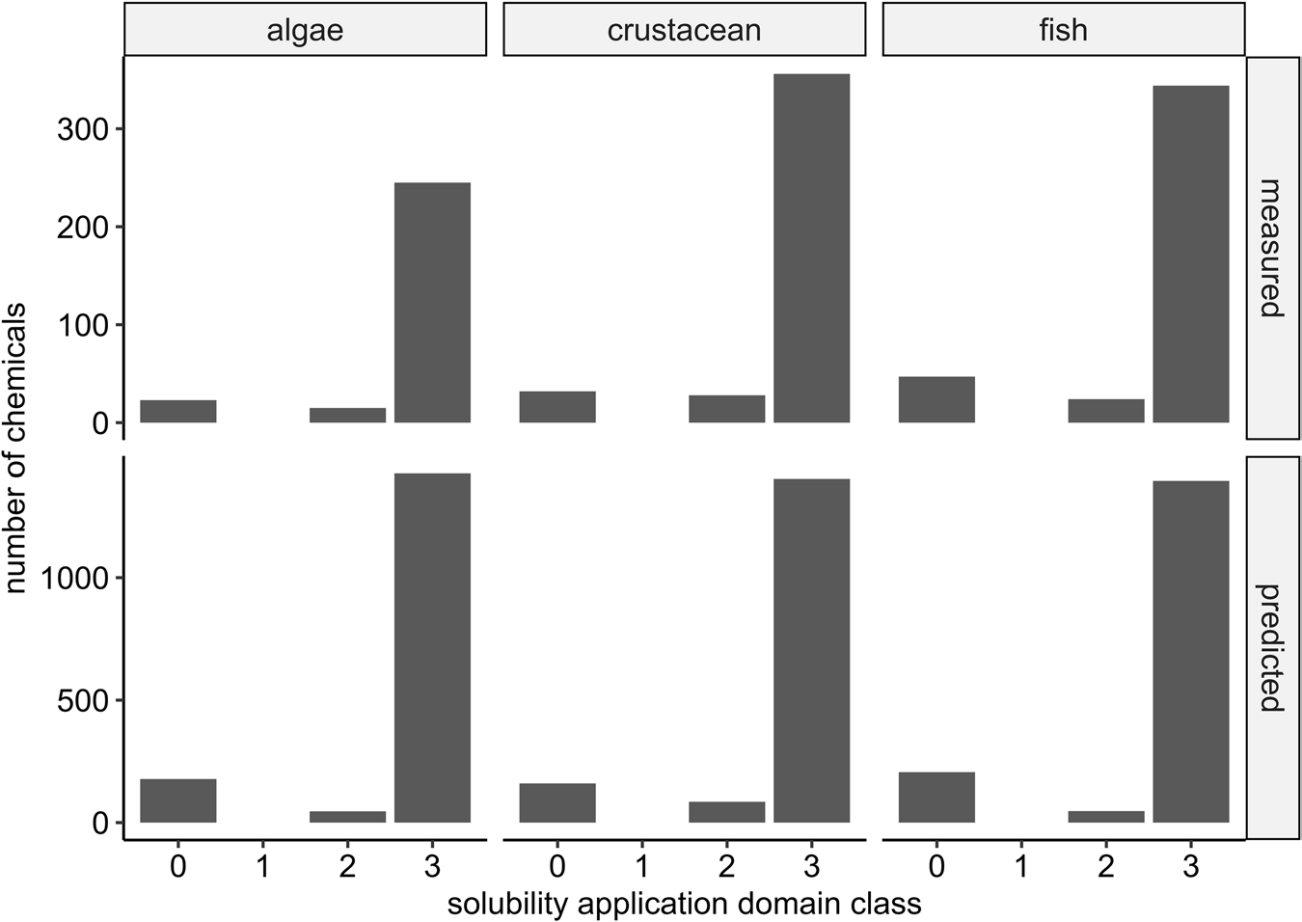
Distribution of solubility domain classes per BQE for all chemicals considered in this dataset with measured and predicted effect concentrations.

To reveal the data quality of the measured and predicted data, we calculated the solubility-based application domain classes based on the ideas realized in ChemProp^45^. In theory, a chemical cannot be tested above its intrinsic water solubility in bioassays. In older studies, only the nominal concentrations (the initially dosed concentrations), but not the real concentrations in the test media, were reported. Thus, the nominal effect concentration may overestimate the effect if the effect value is above the solubility or saturation of the chemical in the test medium. Though, in cases, a solvation agent was used, or the test medium improves solvation, the solubility can be enhanced. Neither the ECOTOXDB nor most of the QSARs address the solubility issue comprehensively. Figure 5 shows the number of chemicals of our dataset within respective solubility domain classes. The majority of chemicals fall into class 3. This means that the determined effect concentrations are below the calculated solubility limits, and, therefore, valid to be used. Class 2 refers to effect concentrations that lay between the solubility limit and a half log step above. Class 2 compounds can be considered valid according to the solvation criterion mentioned above. No chemicals fall into class 1 and only few into class 0. The latter values should be maintained with caution and considered in cases where such chemicals are high-ranking as toxicity drivers in risk assessments based on QSAR predictions. Such effect concentrations might only be valid after experimental confirmations.

### Curated modes of chemical action in comparison to pre-existing data

**Fig. 6:**
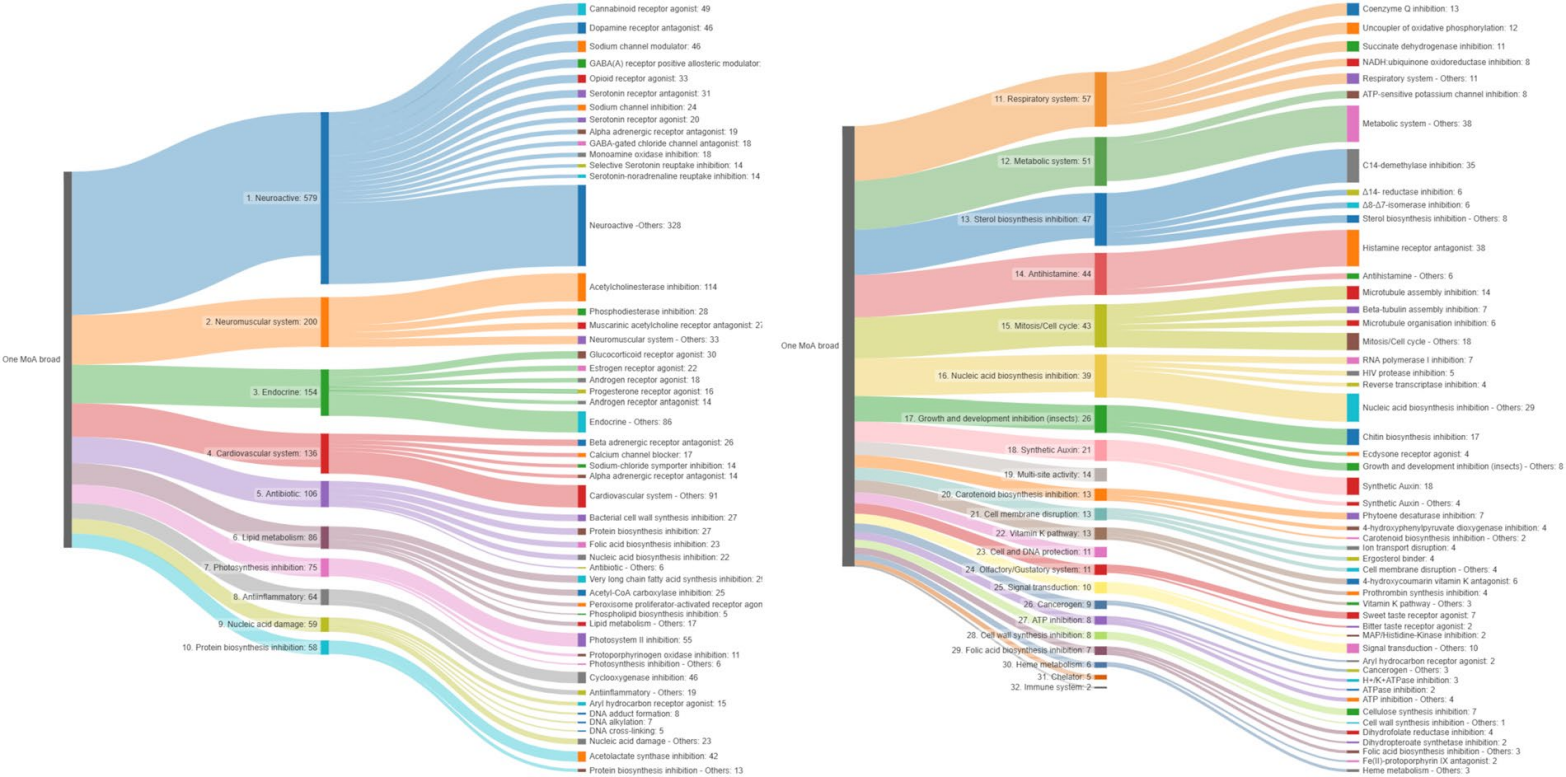
Overview of the 32 broad MoA categories, with their specific sub-categories sorted according to numbers of chemicals within a category. This figure was created using the “SankeyMatic” online tool (https://sankeymatic.com, accessed 15 May 2023).

Modes of chemical action were defined and categorized differently during the last decades. While Verhaar *et al*.^17^ proposed four categories, namely, ‘inert’, ‘less inert’, ‘reactive’, and ‘specifically acting’, based on the composition of chemical structures, later approaches included more categories that were either of general nature (e.g., ‘narcotic action’) or more specifically related to the interaction with a certain biological enzyme or receptor (e.g., ‘acetylcholinesterase inhibition’)^49,50^. In this study, we did not build categories upfront but summarized the existing knowledge into meaningful groups based on biological modes of action. These groups, the broad and specific MoA categories as well as use groups were harmonized and standardized in their names. Duplicates and typos were removed, and harmonization was cross-checked by counting the number of chemicals per term. With this, we provide the largest and most systematic dataset on MoA categories for environmental chemicals. Figure 6 illustrates this by showing all 32 broad MoA categories with respective specific categories within. The comprehensiveness was confirmed by comparing chemicals and assigned MoA categories with results obtained with the tool provided by Firman *et al*.^51^. We found that some MoAs are predicted correctly for many chemicals, e.g., ‘acetylcholinesterase inhibition’ or ‘fatty acid biosynthesis inhibition’ while other MoAs such as interactions of chemicals with endocrine nuclear receptors or biological structures of the nervous system are not well covered by the prediction tool. Hence, the here provided data and the resulting MoA categories can be used to extend tools and approaches that aim to predict MoAs based on chemical structure information as summarized in Firman *et al*.^51^ and Kienzler *et al*.^52^. In turn, the mentioned tools might be applied and evidence generated, especially for those chemicals for which no MoA could be assigned in this study.

## Usage Notes

Data can be used with each software that reads csv. CSV can be imported to excel via the “data/import csv function”.

### Recommendations for the use of the dataset

The dataset was carefully compiled and curated. Quality measures were applied to amend the dataset with quality control tags (i.e. the application domains) to ensure a good data quality and re-usability. The provided dataset should be applied in a scientific and/or regulatory context by experienced assessors or under supervision of an experienced person. To our knowledge, this is the first curation of its kind which provides a starting point and structure that could and should be updated repeatedly as experimental data as well as prediction models constantly evolve^53^. The provided workflow and dataset fulfil the FAIR principles^31^ and therefore enable comprehensive and transparent risk assessments with harmonized data for the aquatic environment as full transparency and reproducibility was not given in so far published large-scale chemical risk assessments for aquatic environments (e.g., Malaji *et al*.^54^, Rorije *et al*.^55^). Furthermore, next to the identification of risk driving compounds or sites at risk due to mixture exposures, the comparison of the available effect data with environmental monitoring data can also provide guidance for the prioritization of chemicals for toxicity testing.

The curated collection of MoAs represents the state of knowledge and is definitely not complete but provides guidance and evidence for risk assessors and scientists. Chemicals can be grouped according to their similar ways of action, novel compounds might be assigned to MoAs via read-across approaches, and machine learning or artificial intelligence approaches might use the data for model training. This means, the collection of MoAs and molecular biological targets of chemicals provided in this study provides guidance for the development of *in vitro* and *in silico* methods that should be applied to identify MoAs of novel future chemicals in high throughput screening approaches prior to animal testing. In this regard, especially methods for the detection of MoAs indicative of chronic toxicity, such as neuroactivity or endocrine action of chemicals need to be further developed as we show here that a substantial number of environmentally relevant chemicals can act respectively. The date collected in this study also indicates that chemicals of diverse use groups can act via the same MoA. However, so far, joint exposures to chemicals of the same use group or with the same MoA belonging to different regulatory silos (legislative sectors) are not considered in risk assessments. Currently, initiatives are under way to change this and consider chemicals in common assessment groups or introduce risk factors for mixture exposures^27,30,56^. The former approach, proposed by EFSA, aims to apply hazard-driven criteria for grouping of chemicals into assessment groups using MoA information on toxicity^27^. Furthermore, ECHA applies MoA information within risk assessments in the frame of read-across and grouping of chemicals^28^. The here provided dataset supports all of these efforts as it can be used i) for read-across studies with novel compounds, ii) for the definition of meaningful MoAs and MoA categories for the grouping of chemicals, and iii) for qualitative and quantitative mixture risk assessments based on data of joint chemical occurrence. Future efforts need to integrate data on more species as are currently curated, e.g., within the EU project PrecisionTox^57^, and further harmonize environmental and human risk assessment frameworks across regulatory silos as it is anticipated, e.g., in the European Partnership for the Assessment of Risks of Chemicals (PARC)^58^.

### Limitations of the dataset

MoA information was curated and researched systematically by using external data sources. For many chemicals, no MoA information was found in those sources, which does not mean that this information is not known or provided elsewhere. We tried to consider MoA knowledge for more than one species group. However, each chemical may act via other MoAs in other species that were not considered here. To our knowledge, there is no ontology for MoAs available and annotations of MoAs are less harmonized than biological molecules, for example. We tried to start MoA annotations considering existing knowledge. However, this dataset is far from being complete and, dependent on the context, the grouping of chemicals into use groups and MoA categories could also be done in other ways.

Despite the careful curation of the ecotoxicity dataset, the input data was retrieved from an external database or QSAR estimations. The underlying data might contain data with quality issues (e.g., bad experimental design, or wrong rating due to reported nominal concentrations). Furthermore, in some cases, only a few data points are available, and thus the predictive power is lacking due to the scarcity of data. This also applies to QSAR models because a limited number of chemicals with similar structural features in the training dataset results in low reliability of the predictions.

### Peer review

The data descriptor was peer reviewed in 2023 based on the data version 2 available on Zenodo^37^.

## Data Availability

The R package REcoTox^43^ version 0.4.1 for processing US EPA ECOTOX Knowledgebase files is available on GitHub (https://github.com/tsufz/REcoTox/releases/tag/0.4.1) under AGPL-3.0 license. The REcoTox and graphics processing scripts are available on Zenodo^44^ under AGPL-3.0 license.
